# Factors Influencing Recurrence Following Transabdominal Preperitoneal Repair for Inguinal Hernias

**DOI:** 10.7759/cureus.50975

**Published:** 2023-12-22

**Authors:** Ainul Hadi, Saadia Muhammad, Muhammad Alam

**Affiliations:** 1 Department of General Surgery, Hayatabad Medical Complex Peshawar, Peshawar, PAK

**Keywords:** transabdominal preperitoneal repair, mesh, myopectineal orifice, recurrence, inguinal hernia

## Abstract

Background: Inguinal hernia repair, a globally prevalent surgical procedure, has witnessed the widespread adoption of the transabdominal preperitoneal (TAPP) approach, promising reduced postoperative pain, faster recovery, and lower recurrence rates. However, hernia recurrence remains a persistent challenge, necessitating a comprehensive exploration of influencing factors specific to TAPP repair.

Objective: The objective of this study is to identify and analyze key factors contributing to inguinal hernia recurrence post-TAPP repair

Materials and methods: This is a retrospective cohort study involving 152 patients undergoing TAPP repair at Hayatabad Medical Complex Peshawar, from November 2020 to October 2023. Demographic, preoperative, and postoperative data were meticulously collected. Statistical analysis employed IBM SPSS Statistics for Windows, Version 25 (Released 2017; IBM Corp., Armonk, New York, United States).

Results: An 8.5% recurrence rate was observed, with influencing factors including insufficient myopectineal orifice (MPO) dissection in three cases (23%), smaller mesh size in two cases (15.4%), infected mesh in two cases (15.4%), mesh dislodgement in one case (20%), and missed sac in one case (20%). Clinical examination and imaging studies diagnosed recurrences in 30.8% and 69.2% of cases, respectively.

Conclusion: Despite the advantages of TAPP repair, hernia recurrence persists, emphasizing the need for ongoing refinement of surgical techniques. This study identifies critical influencing factors, notably insufficient MPO dissection, guiding efforts to enhance the efficacy of TAPP repair and improve patient outcomes.

## Introduction

Inguinal hernia repair is one of the most commonly performed surgical procedures worldwide, with millions of cases reported annually [1]. Among the various surgical techniques employed for inguinal hernia repair, the transabdominal preperitoneal (TAPP) approach has gained significant popularity due to its potential advantages, such as reduced postoperative pain, faster recovery, and lower recurrence rates [2]. Recurrence of inguinal hernias remains a challenging issue in the field of hernia surgery, prompting a critical examination of the factors influencing the recurrence rates in TAPP repair [3].

TAPP repair has evolved as a prominent technique in inguinal hernia management since its introduction. Originating from the principles of laparoscopic surgery, TAPP involves the creation of a preperitoneal space to place the mesh, providing a comprehensive approach to hernia repair. The laparoscopic nature of TAPP allows for improved visualization, reduced tissue trauma, and enhanced precision during the repair process [4].

Numerous studies have highlighted the potential advantages of TAPP repair over traditional open techniques. These include decreased postoperative pain, earlier return to normal activities, and reduced hospital stay [5,6]. However, despite these benefits, the recurrence of inguinal hernias after TAPP repair remains a persistent concern, prompting a thorough investigation into the factors contributing to this phenomenon.

Recurrence in TAPP repair is a multifactorial outcome influenced by various patient-related, surgical, and anatomical factors. Patient-related factors may include age, comorbidities, and overall health status [7]. Surgical factors encompass technique-related nuances, such as mesh placement, fixation methods, and the surgeon's experience. Anatomical considerations, including the type and size of the hernia, also play a crucial role in influencing recurrence rates [8].

Despite the widespread adoption of TAPP repair and the growing body of literature on inguinal hernia outcomes, there exists a notable research gap in comprehensively understanding the factors contributing to recurrence specifically in the TAPP approach [9]. Addressing this gap is essential for optimizing patient outcomes, refining surgical techniques, and informing evidence-based practice. This research article aims to critically review and analyze the factors influencing recurrence in TAPP repair for inguinal hernia.

## Materials and methods

This retrospective cohort study was conducted at the Department of General Surgery, Hayatabad Medical Complex, Peshawar, spanning from November 2020 to October 2023. A total of 208 TAPP procedures were carried out during the study period, 56 patients were excluded due to insufficient medical records, and thus a total of 152 cases who underwent TAPP for inguinal hernias were enrolled. Cases were selected based on the availability of complete medical records. All the TAPP procedures were performed by different consultants of the general surgery department with experience of 07 years or above. Our unit has a predetermined policy for the systematic assessment of patients at regular intervals. The fixed policy entails a follow-up period of up to 12 months, with scheduled assessments at 10 days, 1, 3, 6, and 12 months postoperatively. Patients were diligently followed up during these specified intervals, allowing for comprehensive monitoring of their postoperative course and the identification of any recurrence of inguinal hernias. It is crucial to highlight that patients whose follow-up medical records were unavailable for any of these scheduled assessments were excluded from the study to ensure data completeness and reliability. The exclusion criteria specifically targeted patients with incomplete records or those lost to follow-up during the predefined intervals. This approach was essential to maintain the integrity and accuracy of the study findings.

The surgical technique employed three ports, with the initial 10 mm port above the umbilicus for pneumoperitoneum. A telescope facilitated peritoneal cavity inspection, and two 5-mm ports were strategically placed on either side lateral to the rectus sheath. A peritoneal incision was executed 3-4 cm above the deep ring using an endoscopic scissor with monopolar cautery, followed by sac dissection and creation of the preperitoneal space. In addressing the hernia, a 10×15 polypropylene mesh was inserted and secured with vicryl 2/0. Closure involved continuous sutures of vicryl 2/0 for the peritoneal flap and prolene 2/0 for the skin.

Statistical analysis, performed using IBM SPSS Statistics for Windows, Version 25 (Released 2017; IBM Corp., Armonk, New York, United States), employed descriptive statistics for demographic and clinical characteristics, presenting continuous variables as means or medians and categorical variables as frequencies. The association between various factors and hernia recurrence was assessed through a chi-square test, considering p-values ≤0.05 as statistically significant. Ethical approval was obtained from the Hospital Research and Ethical Committee of Hayatabad Medical Complex under reference #1225. All procedures adhered to the principles outlined in the Declaration of Helsinki, and informed consent was obtained from patients for the use of their medical data in this research.

## Results

A total of 208 TAPP procedures were carried out during the study period, 56 patients were excluded due to insufficient medical records, and thus a total of 152 cases were enrolled. The mean age of the cohort was ±45 years, with a range of 20-70 years. The age range was as follows. Forty-two cases (27.6%) belong to the age group of 20-35 years, 61 (40.1%) belong to the age group of 36-50 years, and 49 (32.2%) belong to the age group of 51-70 years. There were 141 (92.8%) males and 11 (7.2%) females with a male-to-female ratio of 12.8:1.

According to BMI categories, 22(15.5%) were in <18.5 kg/m^2^, 51(33.5%) were in 18.5-24.9 kg/m^2^, 46(30.3%) in 25-29 kg/m^2^ and 33 (21.7%) were classified as >30 kg/m^2^. ASA classification revealed that 51 (33.6%) belonged to ASA-I, 69 (45.4%) to ASA-II and 32 (21%) belonged to ASA-III. The right-side primary hernia was present in 71 (46.7%) cases, the left side in 47 (30.9%) cases, and the bilateral hernia was 34 (22.4%). Direct inguinal hernias were 102 (67.1%) while 50 (32.9%) were indirect inguinal hernias (Table 1).

**Table 1:** Demographic data and other characteristics

Demographics	Frequency	Percentage
Age group		
20-35 years	42	27.6%
36-50	61	40.1%
51-70	49	32.2%
Gender		
Male	141	92.8%
Female	11	7.2%
BMI- Status		
<18.5 kg/m^2^	22	15.5%
18.5-24.9 kg/m^2^	51	33.5%
25-29 kg/m^2^	46	30.3%
>30 kg/m^2^	33	21.7%
ASA-Level		
ASA-I	51	33.6%
ASA-II	69	45.4%
ASA-III	32	21%
ASA-IV	0	0%
Hernia types		
Right-side primary hernia	71	46.7%
Left-side primary hernia	47	30.9%
Bilateral hernia	34	22.4%
Direct hernia	102	67.1%
Indirect hernia	50	32.9%

Hernia recurrence was observed in 13 (8.5%) cases. The mean time to recurrence was ±9 months (range 6-12 months). Recurrences were diagnosed through clinical examination in four (30.8%) cases, while nine cases (69.2%) were identified through imaging studies, which include ultrasonography in three (33.3%) cases, computed tomography (CT) scan in five (55.6%) cases, and magnetic resonance imaging (MRI) in one (20%) case (Table 2).

**Table 2:** Diagnosis and imaging modalities

	Frequency	Percentage
Recurrence	13	8.5%
Diagnosis		
Clinical examination	4	30.8%
Imaging studies	9	69.2%
Imaging modalities		
Ultrasonography	3	33.3%
CT scan	5	55.6%
MRI	1	20%

Factors influencing postoperative recurrence following re-do surgery revealed that insufficient dissection of the myopectineal orifice (MPO) was the most common cause in three (23%) cases, followed by smaller mesh in two (15.4%) cases, mesh dislodgement in one case (20%), and missed sac in one (20%) case, while infected mesh in two (15.4%) cases was identified through clinical findings (Table 3). No mortality was observed during the study period.

**Table 3:** Influencing factors of recurrence MPO: Myopectineal orifice

Factor	Frequency	Percentage
MPO	3	23%
Small size mesh	2	15.4%
Infected mesh	2	15.4%
Mish dislodgement	1	20%
Missed sac	1	20%

## Discussion

Inguinal hernia repair, a frequently performed surgical procedure globally, has seen the rising popularity of the TAPP approach owing to its potential benefits such as reduced postoperative pain and faster recovery [9,10]. However, despite these advantages, the recurrence of inguinal hernias remains a persistent challenge, necessitating a comprehensive examination of influencing factors. This study contributes to the existing literature by investigating factors influencing recurrence in TAPP repair.

The laparoscopic nature of TAPP repair is designed to offer improved visualization, reduced tissue trauma, and enhanced precision during the repair process [11,12]. While multiple studies emphasize the advantages of TAPP repair over traditional open techniques, the recurrence of inguinal hernias after TAPP repair prompts an exploration of contributing factors [13,14].

The present study aligns with existing literature gaps, emphasizing the necessity for a comprehensive understanding of factors contributing specifically to recurrence in the TAPP approach. The optimization of patient outcomes and the refinement of surgical techniques hinge on addressing these nuanced factors. This study aims to bridge this gap by critically reviewing and analyzing the factors influencing recurrence in TAPP repair for inguinal hernia.

In our study, we observed a 13 (8.5%) recurrence rate. This finding is consistent with previous studies highlighting the persistent challenge of hernia recurrence even in the laparoscopically advanced TAPP approach [15,16]. The median time to recurrence was ±9 months, reinforcing the importance of prolonged postoperative vigilance.

Diagnosis of recurrences varied, with clinical examination accounting for four (30.8%) cases, and imaging studies including ultrasonography, CT scans, and MRI, identifying nine (69.2%) recurrences. This emphasizes the importance of combining clinical assessment with advanced imaging modalities for a comprehensive evaluation of recurrence.

After a re-do surgery for hernia recurrence, our analysis of influencing factors revealed that insufficient dissection of the MPO emerged as the most common cause of recurrence 3(23%). This underscores the critical role of the meticulous surgical technique in preventing recurrences. Other factors, including a smaller mesh size, mesh dislodgement, and missed sac, further contribute to the multifactorial nature of recurrence in TAPP repair.

The study's single-center design may limit generalizability, underscoring the necessity for multicenter investigations. The 12-month follow-up might miss late recurrences, emphasizing the need for extended observation periods. While the 152-patient sample is suitable for specific analyses, enlarging it could enhance statistical power. Exclusion criteria, like incomplete records, may introduce selection bias, cautioning against generalizing to diverse populations. Surgeon-dependent variability in technique poses challenges in quantifying its impact on recurrence rates. Although the study identifies factors like insufficient MPO dissection and smaller mesh size contributing to recurrence, a more in-depth exploration of their interplay is warranted. Despite these limitations, the research forms a foundational understanding of recurrence in TAPP repair, stressing the ongoing need for refining surgical techniques and improving patient outcomes.

## Conclusions

In conclusion, our study on TAPP repair for inguinal hernias revealed a 15.1% recurrence rate within a 12-month follow-up. Factors such as insufficient MPO dissection, a smaller mesh size, infected mesh, dislodged mesh, and missed sac significantly influenced recurrence. Despite TAPP's advantages, the persistence of hernia recurrence highlights the complexity of the issue. This underscores the need for ongoing refinement of surgical techniques to enhance the efficacy of TAPP repair and improve patient outcomes.
